# Overlapping but Disparate Inflammatory and Immunosuppressive Responses to SARS-CoV-2 and Bacterial Sepsis: An Immunological Time Course Analysis

**DOI:** 10.3389/fimmu.2021.792448

**Published:** 2021-12-09

**Authors:** Tyler J. Loftus, Ricardo Ungaro, Marvin Dirain, Philip A. Efron, Monty B. Mazer, Kenneth E. Remy, Richard S. Hotchkiss, Luer Zhong, Rhonda Bacher, Petr Starostik, Lyle L. Moldawer, Scott C. Brakenridge

**Affiliations:** ^1^ Department of Surgery and the Sepsis and Critical Illness Research Center, University of Florida College of Medicine, Gainesville, FL, United States; ^2^ Departments of Anesthesiology and Pediatrics, Washington University in St. Louis School of Medicine, St. Louis, MO, United States; ^3^ Department of Biostatistics, University of Florida College of Medicine, Gainesville, FL, United States; ^4^ Department of Pathology, Immunology and Laboratory Medicine, University of Florida College of Medicine and Shands Hospital-UF Health Science Center, Gainesville, FL, United States

**Keywords:** inflammation, immune suppression, ELISpot, MDSC, immune response

## Abstract

Both severe SARS-CoV-2 infections and bacterial sepsis exhibit an immunological dyscrasia and propensity for secondary infections. The nature of the immunological dyscrasias for these differing etiologies and their time course remain unclear. In this study, thirty hospitalized patients with SARS-CoV-2 infection were compared with ten critically ill patients with bacterial sepsis over 21 days, as well as ten healthy control subjects. Blood was sampled between days 1 and 21 after admission for targeted plasma biomarker analysis, cellular phenotyping, and leukocyte functional analysis *via* enzyme-linked immunospot assay. We found that circulating inflammatory markers were significantly higher early after bacterial sepsis compared with SARS-CoV-2. Both cohorts exhibited profound immune suppression through 21 days (suppressed HLA-DR expression, reduced mononuclear cell IFN-gamma production), and expanded numbers of myeloid-derived suppressor cells (MDSCs). In addition, MDSC expansion and *ex vivo* production of IFN-gamma and TNF-alpha were resolving over time in bacterial sepsis, whereas in SARS-CoV-2, immunosuppression and inflammation were accelerating. Despite less severe initial physiologic derangement, SARS-CoV-2 patients had similar incidence of secondary infections (23% vs 30%) as bacterial sepsis patients. Finally, COVID patients who developed secondary bacterial infections exhibited profound immunosuppression evident by elevated sPD-L1 and depressed HLA-DR. Although both bacterial sepsis and SARS-CoV-2 are associated with inflammation and immune suppression, their immune dyscrasia temporal patterns and clinical outcomes are different. SARS-CoV-2 patients had less severe early inflammation and organ dysfunction but had persistent inflammation and immunosuppression and suffered worse clinical outcomes, especially when SARS-CoV-2 infection was followed by secondary bacterial infection.

## 1 Introduction

Serious SARS-CoV-2 infections manifest many of the classic sequelae of sepsis, including organ injury and immunological dyscrasia (1–3). Whereas the incidence of SARS-CoV-2 infection is projected to decline substantially over time, bacterial sepsis will likely remain the most common cause of post-pandemic in-hospital mortality, morbidity, and healthcare expenditures (4, 5). The substantial burden of long-term physical, cognitive, social, and psychological effects of sepsis and SARS-CoV-2 infection underscore the importance of early diagnosis and effective treatment to prevent progression to multiple organ failure, chronic critical illness (CCI), and the persistent inflammation, immunosuppression, and catabolism syndrome (PICS) (6, 7).

Although early source control, antibiotic therapy, and resuscitation improve clinical outcomes for bacterial sepsis patients, efforts to develop targeted immunotherapies have been hindered by heterogeneous treatment responses among sepsis phenotypes and difficulty identifying, understanding, and modulating adaptive and maladaptive elements of the immune response (8). SARS-CoV-2 infection has similar challenges, but unlike sepsis, has no effective immediate pathogen source control; antiviral therapies and glucocorticoids are only supportive in nature. In addition, SARS-CoV-2 clinical trajectories and the natural history of immunological dyscrasia over time have only been infrequently compared with that of bacterial sepsis, and the purported SARS-CoV-2-related inflammatory cytokine storm remains controversial. A more detailed, time-dependent comparison could confer deeper understanding of dynamic host immunity and its associations with clinical outcomes.

In this prospective cohort study, the inflammatory and immunosuppressive profiles of 30 critically ill patients with SARS-CoV-2 infection were compared with ten critically ill bacterial sepsis patients at five time-points over a 21-day period, and ten healthy control subjects. We hypothesized that SARS-CoV-2 sepsis, which is treated with supportive care rather than curative intent, would be associated with more persistent immune dyscrasia and increased hospital mortality relative to bacterial sepsis, where source control is of immediate concern.

## 2 Materials and Methods

### 2.1 Study Design and Subject Enrollment

This is a prospective, observational, longitudinal sampling study of 30 hospitalized patients with SARS-CoV-2 infection and ten patients with bacterial infection, all of whom were adjudicated as being septic (i.e., evidence of life-threatening organ dysfunction due to infection) per Sepsis-3 guidelines (9). Patients were identified, screened, and enrolled between May and December, 2020 from the intensive care units (ICUs) of Shands Hospital-Gainesville, a tertiary care center in North Central Florida. Patients were enrolled upon ICU admission and within 24 hours of inpatient treatment for SARS-CoV-2 or bacterial infection. Among 892 patients screened, 582 met inclusion criteria and 542 were excluded for the following reasons: enrolled in another study (n=178), prisoner or under guardianship (n=137), age (n=90), exclusionary patient condition (transplant recipient, immunocompromised, malignancy, pregnant, Do Not Resuscitate order, n=114), and declined enrollment (n=23). Ethics approval was obtained from the University of Florida Institutional Review Board (#IRB202000971). Informed consent was obtained from each subject or their surrogate decision-maker. This study was performed in accordance with Strengthening the Reporting of Observational Studies in Epidemiology (STROBE) guidelines, as listed in **Supplemental Table 1**.

Blood samples were collected within 24 hours of inpatient treatment initiation and on days 4, 7, 14, and 21 while patients remained in the ICU. Heparin and EDTA-anticoagulated whole blood were collected at each time point. HLA-DR expression on CD14^+^ cells was measured on days 1, 4 and 7; MDSC numbers and phenotype were also assessed by flow cytometry on days 4 and 14. Due to institutional environmental health and safety regulations regarding aerosolization of SARS-CoV-2 positive biologic specimens during flow cytometry, isolated cells were fixed with buffered formalin prior to flow cytometric analysis. On days 1, 4, and 7, peripheral blood mononuclear cells (PBMC) were isolated from unfixed heparinized whole blood by density centrifugation, and subsequent enzyme-linked immunospot assay (ELISpot) was used to assess *ex vivo* responsiveness by TLR4-expressing monocytes and T-cells. Remaining EDTA whole blood was also centrifuged at 1800 x g at all sampling intervals, and plasma was frozen at -80° C for subsequent cytokine analyses.

Bacterial sepsis patients were managed using sepsis resuscitation bundles that were originally implemented as a computerized clinical decision-support platform and subsequently embedded within electronic health record order sets and clinical note templates (10–12). Briefly, sepsis patients underwent early goal-directed fluid resuscitation, received empiric antibiotic therapy tailored to the presumed anatomic site of infection, and were referred for early source control procedures when appropriate. Patients with SARS-CoV-2 infection meeting Sepsis-3 criteria received similar care by sepsis management bundles with the caveat that intravenous fluid resuscitation could be limited to less than the standard, initial 30 mL/kg intravenous fluid bolus, especially for patients with right heart failure, renal failure, or high risk for hydrostatic pulmonary edema-related respiratory failure (9). During the study time period, subjects received remdesivir and glucocorticoids as standard of care for their clinical management. Mechanical ventilation strategies for patients with SARS-CoV-2 sepsis were tailored to individual patients at the discretion of critical care providers. The occurrence, date, and time of secondary bacterial infections were ascertained from microbiologic data and clinical documentation, including the assessment of board-certified Infectious Disease specialists in determining whether SARS-CoV-2 patients required empiric antibiotic therapy for bacterial pneumonia.

Ten healthy control subjects were matched to SARS-CoV-2 infected subjects by age, gender, and race/ethnicity. A single venous blood sample was obtained from each healthy subject after obtaining informed consent.

### 2.2 Analytical Methods

#### 2.2.1 Plasma Cytokine Analyses

Plasma IL-1-beta, IL-6, IL-8, IL-10, TNF-alpha, G-CSF, MCP-1/CCL2 and soluble programmed death ligand 1 (sPD-L1; B7-H1) were determined by immunoassay using the Luminex Magpix™ platform (Thermo-Fisher, Waltham, MA) and a standard ELISA assay (R&D Sytems, Inc, Minneapolis, MN).

#### 2.2.2 HLA-DR Expression

HLA-DR expression on CD14^+^ cells was analyzed on formalin-fixed, heparinized whole blood using the HLA-DR Quantibrite™ system (Becton-Dickinson, Franklin Lakes, NJ) according to the manufacturer’s instructions.

#### 2.2.3 MDSC Quantitation

Formalin-fixed whole blood was diluted in equal volume of PBS and layered over Ficoll Paque™ PLUS (GE Healthcare, Uppsala, Sweden). The PBMC layer was isolated following centrifugation at 400 x g for 30 min at room temperature. PBMC samples were labeled with CD33^+^ conjugated to APC, CD11b^+^ conjugated to APC-Cy7, HLA-DR conjugated to FITC, CD14^+^ conjugated to Pac-blue, and CD66b^+^ conjugated to brilliant violet (BV421) (all antibodies from Becton-Dickinson). Compensation controls were used to correct for fluorescence spectral overlap. Fluorescence Minus One (FMO) controls were used to optimize the gating strategy and ensure correct cell populations were captured. The samples were analyzed on the ZE5 Cell Analyzer (Bio-Rad, Hercules, CA), and MDSCs were characterized as CD33^+^CD11b^+^HLA-DR^low/−^. Monocytic MDSCs (M-MDSCs) were further characterized as CD14^+^ and granulocytic MDSCs (PMN-MDSCs) as CD14^−^CD66b^+^, while non-monocytic, non-PMN, early progenitor (E-MDSCs) were CD14^−^CD66b^-^ (13). Our gating strategy is illustrated in **Supplemental Figure 1**.

#### 2.2.4 ELISpot Assay

ELISpot was used to measure the production of TNF-alpha and IFN-gamma *ex vivo* in stimulated PBMCs using single-color ELISpot kits (ImmunoSpot; Cellular Technology Limited, Cleveland, OH), and was performed as previously described. Lipopolysaccharide (LPS) from *E. coli*, serotype 055:B5 (ALX-581-013, Enzo Life Sciences, Farmingdale, NY) at a concentration of 1,250 ng/ml was used to induce TNF-alpha expression. 125 ng/mL of anti-human CD3 (clone HIT3a; BioLegend, San Diego, CA) and 1,250 ng/mL of anti-human CD28 (clone CD28·2; BioLegend, San Diego, CA) antibodies were used to induce IFN-gamma expression. Cell concentration of 5 x 10^3^ were plated into each well for LPS and 5 x10^4^ were plated per well for CD3/CD28 stimulation. Both TNF-alpha and IFN-gamma analyses were performed with and without IL-7 stimulation. Samples were scanned and analyzed for spot counts and spot size using ImmunoSpot S6 Entry Analyzer with ImmunoSpot 7·0·30·4 professional software (CTL Analyzers, Cleveland, OH). Spot counts and spot size were reported as spot forming units (SFU) and mm^2^, respectively. Total expression was calculated as the product of the number of SFU and the spot size. Spot counts represent the number of cytokine-producing T cells, spot sizes represent the magnitude of the cytokine secretory response by individual cells, and total expression values represent the magnitude of total cytokine secretion for all cells in the sample. Illustrative examples are provided in **Supplemental Figure 2**.

#### 2.2.5 Statistical Analyses

Descriptive data are presented as frequency and percentage or median and 25th/75th percentiles. Fisher’s exact test was used to compare categorical variables. The Wilcoxon test and generalized estimating equations were used to compare continuous variables at discrete timepoints, and generalized estimating equations were also used to compare variables over time. Given small sample sizes, data missing at random (see **Supplemental Table 2**) were not imputed; all statistical analyses were performed exclusively on available data. All statistical analyses were performed using SAS (v·9·4, Cary, NC, USA). SARS-CoV-2 patients who develop secondary bacterial infections represent a distinct subgroup that would be expected to have inflammatory, immunologic, and clinical outcome profiles blending those of SARS-CoV-2 and bacterial sepsis; therefore, we performed subgroup analyses in which the SARS-CoV-2 cohort was split into a subgroups with and without secondary bacterial infections. Given the lack of prior studies in peer-reviewed journals describing temporal relationships of inflammatory and immunosuppressive pathways in SARS-CoV-2 and bacterial sepsis patients, this was considered an exploratory study performed without a power analysis and sample size was determined by gestalt. Despite the exploratory nature of this study, due to the large number of statistical comparisons of plasma cytokines, HLA-DR expression values, MDSC quantities, and ELISpot assay values, p-value adjustments were performed for each of these analyses using the Benjamini-Hochberg procedure. Univariate associations between hospital mortality and early indicators of inflammation, immune suppression, and MDSC populations were assessed by logistic regression without adjustment for multiple comparisons. All significance tests were two-sided, with a p-value <0·05 considered statistically significant.

## 3 Results

Thirty clinically adjudicated SARS-CoV-2 infected and ten bacterial sepsis hospitalized patients were enrolled, along with ten healthy control subjects. As shown in **Table 1**, the SARS-CoV-2 and bacterial sepsis patients did not differ by age, biologic sex, race, individual major comorbidities, Charlson comorbidity index, or body mass index. However, bacterial sepsis subjects had significantly higher initial physiologic derangement evident by higher APACHEII scores and incidence of receiving vasopressor and mechanical ventilation support. Seven of the 30 SARS-CoV-2 patients developed secondary bacterial infections. Five SARS-CoV-2 patients died (17%), while none of the ten bacterial sepsis patients died. The incidence of hospital readmission within 6 months was greater in the bacterial sepsis group; other 6- and 12-month outcomes, including mortality, were similar between groups.

**Table 1:** Characteristics of SARS-CoV-2, bacterial sepsis, and healthy control cohorts.

Patient characteristics	SARS-CoV-2 n = 30	Bacterial Sepsis n = 10	Healthy Controls n = 10	*p^a^*	*p^b^*	*p^c^*
Age, median [IQR]	54.0 [48.8-63.2]	52.5 [43.0-57.0]	45.5 [38.5-51.5]	0.50	**0.03**	0.26
Female, n (%)	11 (36.7)	5 (50.0)	4 (40.0)	0.48	>0.99	>0.99
Race, n (%)						
African American	9 (30.0)	1 (10.0)	1 (10.0)	0.40	0.40	>0.99
American Indian	0 (0.0)	0 (0.0)	0 (0.0)	>0.99	>0.99	>0.99
Asian	0 (0.0)	0 (0.0)	0 (0.0)	>0.99	>0.99	>0.99
Pacific Islander	0 (0.0)	0 (0.0)	0 (0.0)	>0.99	>0.99	>0.99
White	19 (63.3)	9 (90.0)	9 (90.0)	0.23	0.23	>0.99
Other or Unknown	2 (6.7)	0 (0.0)	0 (0.0)	>0.99	>0.99	>0.99
Body mass index, kg/m^2^, median [IQR]	35.2 [32.6-41.5]	30.8 [26.7-50.1]	–	0.66	–	–
Charlson comorbidity index, median [IQR]	2.0 [0.2-3.8]	1.5 [1.0-2.0]	0.0 [0.0-0.0]	0.54	**<0.01**	**<0.01**
Comorbidities, n (%)						
Acute renal failure	3 (10.0)	0 (0.0)	0 (0.0)	0.56	0.56	>0.99
Chronic obstructive pulmonary disease	0 (0.0)	0 (0.0)	0 (0.0)	>0.99	>0.99	>0.99
Congestive heart failure	1 (3.3)	0 (0.0)	0 (0.0)	>0.99	>0.99	>0.99
Current smoker	1 (3.3)	1 (10.0)	0 (0.0)	0.44	>0.99	>0.99
Diabetes	17 (56.7)	3 (30.0)	0 (0.0)	0.27	**<0.01**	0.21
Oral hypoglycemic prescription	15 (50.0)	1 (10.0)	0 (0.0)	**0.03**	**<0.01**	>0.99
Insulin prescription	6 (20.0)	0 (0.0)	0 (0.0)	0.31	0.31	>0.99
Dialysis dependent	0 (0.0)	0 (0.0)	0 (0.0)	>0.99	>0.99	>0.99
Disseminated cancer	0 (0.0)	0 (0.0)	0 (0.0)	>0.99	>0.99	>0.99
Hypertension	17 (56.7)	4 (40.0)	0 (0.0)	0.47	**<0.01**	0.09
Myocardial infarction	0 (0.0)	0 (0.0)	0 (0.0)	>0.99	>0.99	>0.99
Peripheral vascular disease	0 (0.0)	0 (0.0)	0 (0.0)	>0.99	>0.99	>0.99
Steroid use	0 (0.0)	0 (0.0)	0 (0.0)	>0.99	>0.99	>0.99
>10% weight loss in prior 6 months	0 (0.0)	0 (0.0)	0 (0.0)	>0.99	>0.99	>0.99
Illness severity						
APACHE II score^d^, median [IQR]	7.5 [4.2-15.8]	13.5 [12.2-17.5]	**-**	**0.02**	**-**	**-**
MEWS score^d^, median [IQR]	3.0 [2.0-4.0]	5.0 [4.0-6.0]	**-**	**0.01**	**-**	**-**
Received vasopressors, n (%)	2 (6.7)	6 (60.0)	**-**	**<0.01**	**-**	**-**
Received mechanical ventilation, n (%)	4 (13.3)	6 (60.0)	**-**	**<0.01**	**-**	**-**

IQR, interquartile range; APACHE, Acute Physiology and Chronic Health Evaluation; MEWS, modified early warning score. P-values represent group comparisons for each variable in the “Patient characteristics” column. Fisher’s exact test was used to compare categorical variables. The Kruskal–Wallis test was used to compare continuous variables.

a SARS-CoV-2 vs. Bacterial Sepsis.

b SARS-CoV-2 vs. Healthy Controls.

c Bacterial Sepsis vs. Healthy Controls.

d At the time of diagnosis with SARS-CoV-2 or bacterial sepsis.

Bold values indicate p<0.05, which was our threshold for statistical significance.

### 3.1 Inflammation

**Figure 1:**
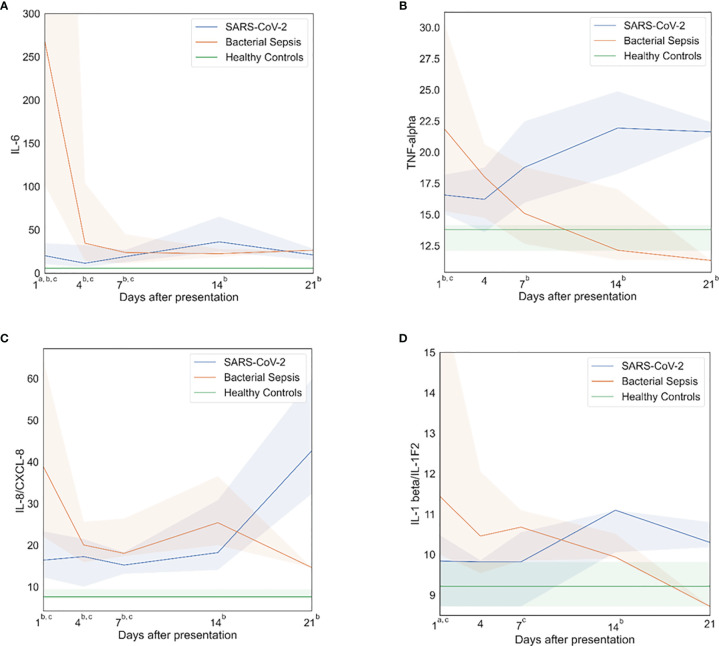
Inflammatory profiles. After bacterial sepsis, inflammatory cytokines peaked on sampling day one and then declined; after SARS-CoV-2 infection, inflammatory cytokines were persistently elevated or increased over time. **(A)** Interleukin (IL)-6 levels over time. **(B)** Tumor necrosis factor (TNF)-alpha levels over time. **(C)** IL-8 levels over time. **(D)** IL-1 beta levels over time. Data are presented as median values (colored lines) and interquartile ranges (shaded regions). Groups were compared by generalized estimating equations with p-values corrected for multiple comparisons by the Benjamini-Hochberg procedure. At each time point, all available values (as listed in **Supplemental Table 2**) were included in all statistical tests for 30 SARS-CoV-2 patients, 10 bacterial sepsis patients, and 10 healthy control patients. Superscript letters indicate time points at which group comparisons had p≤0.05. a SARS-CoV-2 vs. Bacterial Sepsis; b SARS-CoV-2 vs. Healthy Controls; c Bacterial Sepsis vs. Healthy Controls.

Plasma cytokines (IL-1-beta, IL-6, IL-8, IL-10, TNF-alpha, G-CSF and MCP-1/CCL2) were measured as biomarkers of inflammation. Both SARS-CoV-2 and bacterial sepsis patients had significantly elevated levels of inflammatory cytokines when compared with healthy subjects, but the pattern, magnitude and timing differed significantly, as illustrated in **Figure 1** and **Supplemental Figure 3**. Among bacterial sepsis patients, most cytokine concentrations peaked at the first sampling and declined progressively thereafter. IL-6 (**Figure 1A**) and IL-1-beta (**Figure 1D**) concentrations were both significantly higher on day 1 in bacterial sepsis compared with SARS-CoV-2. After day 1, all inflammatory cytokine concentrations were similar between bacterial sepsis and SARS-CoV-2. In the bacterial sepsis group, TNF-alpha (**Figure 1B**) decreased over time, as examined by a generalized estimating equation (p=0·03); other inflammatory markers also decreased somewhat over time, though the differences were not statistically significant. In contrast, SARS-CoV-2 sepsis patients had cytokine concentrations that were elevated versus healthy control subjects during the entire study period, and remained constant or increased during ICU admission, especially TNF-alpha. In the SARS-CoV-2 group, TNF-alpha increased significantly over time, as tested by a generalized estimating equation (**Figure 1B**, p<0·01). There were no significant associations between hospital mortality and day 1 indicators of inflammation, as listed in **Supplemental Table 3**.

### 3.2 Immune Suppression

**Figure 2:**
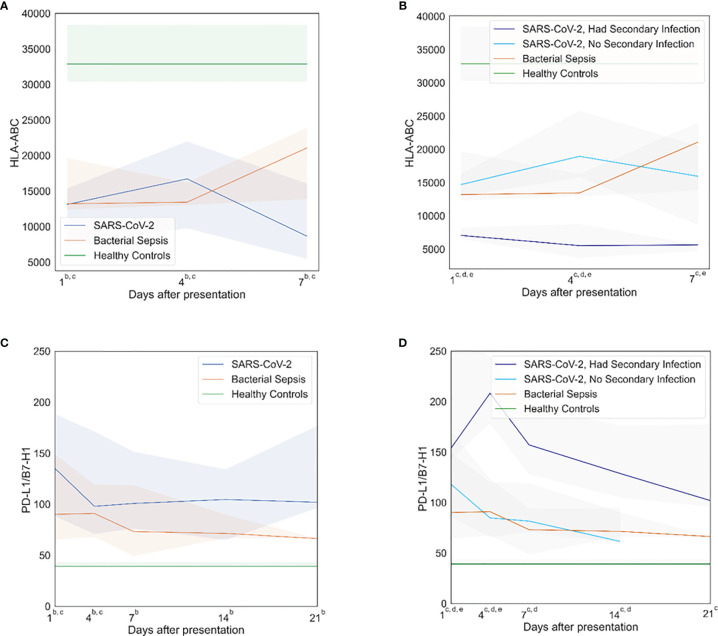
Immunosuppressive profiles. Both SARS-CoV-2 and bacterial sepsis were associated with early, persistent immune suppression. SARS-CoV-2 patients who develop secondary bacterial infections represent a distinct subgroup that would be expected to have immunologic profiles blending those of SARS-CoV-2 and bacterial sepsis. Subgroup analyses demonstrated that HLA-DR expression was lowest and sPD-L1 were highest among SARS-CoV-2 who developed secondary bacterial infections. **(A)** Human leukocyte antigen (HLA)-antibodies bound per cell (ABC) over time, primary analysis. **(B)** HLA-ABC over time, subgroup analysis. **(C)** Programmed death-ligand (PD-L)-1 over time, primary analysis. **(D)** PDL-1 over time, subgroup analysis. Data are presented as median values (colored lines) and interquartile ranges (shaded regions). Groups were compared by generalized estimating equations with p-values corrected for multiple comparisons by the Benjamini-Hochberg procedure. At each time point, all available values (as listed in **Supplemental Table 2**) were included in all statistical tests for 30 SARS-CoV-2 patients, 10 bacterial sepsis patients, and 10 healthy control patients. Superscript letters indicate time points at which group comparisons had p≤0.05. For the primary analysis (3 groups): a SARS-CoV-2 vs. Bacterial Sepsis; b SARS-CoV-2 vs. Healthy Controls; c Bacterial Sepsis vs. Healthy Controls. For the subgroup analysis (4 groups): a SARS-CoV-2 with secondary infection vs. Bacterial Sepsis; b SARS-CoV-2 without secondary infection vs. Bacterial Sepsis; c SARS-CoV-2 with secondary infection vs. Healthy Controls; d SARS-CoV-2 without secondary infection vs. Healthy Controls; eBacterial Sepsis vs. Healthy Controls.

Both SARS-CoV-2 and bacterial sepsis patients showed dramatic evidence of immune suppression, early (within 1 day) and persistently throughout hospitalization. In several cases, the responses were similar between SARS-CoV-2 and bacterial sepsis subjects. HLA-DR expression on CD14^+^ cells was similarly reduced in both sepsis cohorts at days 1, 4, and 7 but was trending back to normal by day 7 in the bacterial sepsis group, while declining further in the SARS-CoV-2 cohort, as illustrated in **Figure 2A**. Similar significant differences and temporal patterns were seen in plasma sPD-L1 concentrations (**Figure 2C**). Subgroup analyses demonstrated that HLA-DR expression (**Figure 2B**) was lowest and sPD-L1 (**Figure 2D**) were highest among SARS-CoV-2 who developed secondary bacterial infections at all time points. These results must be interpreted in the context that median time to secondary infection in SARS-CoV-2 patients was 14 days (**Table 2**).

**Table 2:** Clinical outcomes for patients with SARS-CoV-2 or bacterial sepsis.

Patient outcomes	SARS-CoV-2 n = 30	Bacterial Sepsis n = 10	*p*
Secondary infection, n (%)	7 (23.3)	3 (30.0)	0.69
Pulmonary infection	6 (20.0)	3 (30.0)	0.67
Bloodstream infection	1 (3.3)	0 (0.0)	>0.99
Skin or soft tissue infection	0 (0.0)	1 (10.0)	0.25
Non-infectious complication, n (%)	9 (30.0)	5 (50.0)	0.28
Days between onset and secondary infection, median [IQR]	14.7 [7.1-97.2]	3.8 [2.0-5.2]	0.14
ICU length of stay, days, median [IQR]	0.0 [0.0-8.8]	8.0 [7.0-11.8]	**0.03**
ICU length of stay ≥14 days, n (%)	6 (20.0)	2 (20.0)	>0.99
ICU-free days, median [IQR]	6.0 [5.0-8.0]	5.0 [3.0-6.8]	0.33
Ventilator days, median [IQR]	0.0 [0.0-5.2]	2.5 [1.2-4.5]	0.14
Hospital length of stay, days, median [IQR]	11.0 [6.0-16.0]	13.5 [10.0-18.0]	0.23
Discharge disposition, n (%)			
Hospital mortality	5 (16.7)	0 (0.0)	0.31
Home	19 (63.3)	7 (70.0)	>0.99
Hospice	2 (6.7)	0 (0.0)	>0.99
Inpatient rehabilitation	1 (3.3)	1 (10.0)	0.44
Left against medical advice	1 (3.3)	0 (0.0)	>0.99
Long term acute care	1 (3.3)	1 (10.0)	0.44
Skilled nursing facility	1 (3.3)	1 (10.0)	0.44
Poor discharge disposition,^a^ n (%)	9 (30.0)	2 (20.0)	0.70
Chronic critical illness,^b^ n (%)	3 (10.0)	1 (10.0)	>0.99
Adverse clinical outcome,^c^ n (%)	10 (33.3)	5 (50.0)	0.46
Discharge to 6-month follow-up, n (%)			
Readmission	1 (3.3)	4 (40.0)	**0.01**
Infection without readmission	0 (0.0)	1 (10.0)	0.27
Death	3 (10.0)	1 (10.0)	>0.99
6-12-month follow-up, n (%)			
Readmission	1 (3.3)	0 (0.0)	>0.99
Infection without readmission	1 (3.3)	0 (0.0)	>0.99
Death	0 (0.0)	0 (0.0)	>0.99

P-values represent group comparisons for each variable in the “Patient characteristics” column. Fisher’s exact test was used to compare categorical variables. The Kruskal–Wallis test was used to compare continuous variables.

a Long-term acute care, skilled nursing facility, hospice, or hospital mortality.

b ICU length of stay 14 days or greater with sequential organ failure assessment score 2 or greater on day 14.

c Poor discharge disposition, chronic critical illness, or secondary infection.

Bold values indicate p<0.05, which was our threshold for statistical significance.

**Figure 3:**
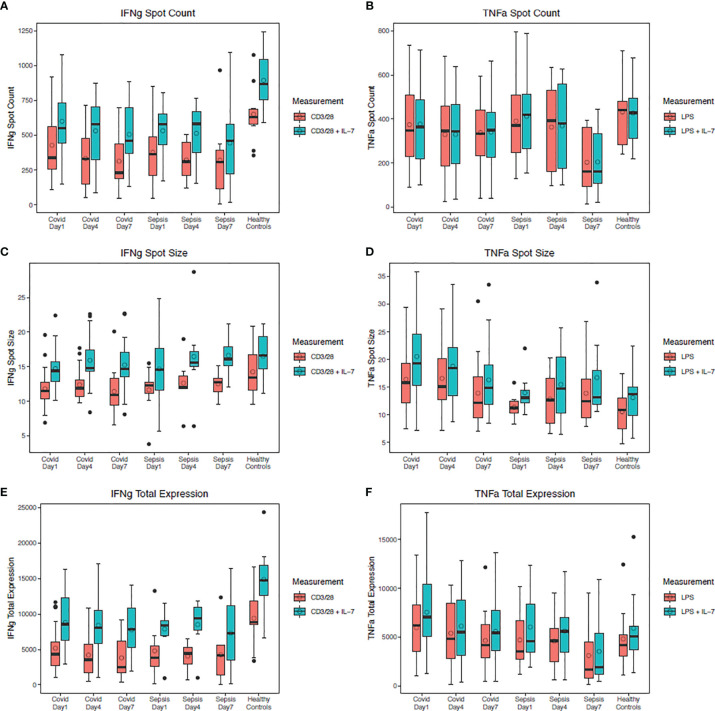
TNF-alpha and IFN-gamma production in stimulated peripheral blood mononuclear cells. Four days after SARS-CoV-2 or bacterial sepsis, interferon (IFN)-gamma production in response to antiCD3/CD28 with IL-7 stimulation was significantly reduced compared with healthy controls (p=0.02 and p=0.04, respectively). Tumor necrosis factor (TNF)-alpha production in response to LPS stimulation with and without IL-7 stimulation was similar among all groups at all time points (all p>0.05). **(A)** IFN-gamma spot count over time with and without IL-7 stimulation. **(B)** TNF-alpha spot count over time with and without IL-7 stimulation. **(C)** IFN-gamma spot size over time with and without IL-7 stimulation. **(D)** TNF-alpha spot size over time with and without IL-7 stimulation. **(E)** IFN-gamma total expression over time with and without IL-7 stimulation. **(F)** TNF total expression over time with and without IL-7 stimulation. Data are presented as median values (bars) with interquartile ranges (boxes), mean values (circles within boxes), maximum and minimum values excluding outliers (whiskers), and outliers (circles outside whiskers), with outliers defined as being 1.5 times the interquartile range beyond the 25th or 75th percentile. Groups were compared by generalized estimating equations with p-values corrected for multiple comparisons by the Benjamini-Hochberg procedure. At each time point, all available values (as listed in **Supplemental Table 2**) were included in all statistical tests for 30 SARS-CoV-2 patients, 10 bacterial sepsis patients, and 10 healthy control patients. D, day; CD, cluster of differentiation; IL, interleukin.

The *ex vivo* responsiveness of PBMCs to TLR4- and T cell-receptor activation, as measured by ELISpot quantification of both TNF-alpha and IFN-gamma respectively, differed between SARS-CoV-2 and bacterial sepsis patients. *Ex vivo* PBMC production of IFN-gamma in response to anti-CD3/CD28 was used as a metric of T-cell or adaptive immune responsiveness, while TNF-alpha production in response to TLR4 stimulus (LPS) was used as a metric of innate immune or inflammatory cell responsiveness (1, 14). In both SARS-CoV-2 and bacterial sepsis patients, total IFN-gamma production in response to antiCD3/CD28 with IL-7 stimulation was significantly reduced compared with healthy subjects on days 4 and 7 (all p ≤ 0·05), as illustrated in **Figure 3**. This was manifest predominantly in both groups by a reduced number of IFN-gamma-producing cells, as measured by spot numbers (**Figure 3A**). Interestingly, only in the SARS-CoV-2 group did the decrease in the number of IFN-gamma-producing cells and total IFN-gamma expression consistently decline over time, with greatest decline among SARS-CoV-2 patients with secondary infection (spot count p<0·01, total expression p=0·03). Total *ex vivo* expression of TNF-alpha in response to LPS stimulation with and without IL-7 stimulation was similar among all groups at all time points (**Figure 3F**, all p>0·05). In the bacterial sepsis and SARS-CoV-2 with secondary infection groups, TNF-alpha expression with LPS and IL-7 stimulation decreased significantly over time, as tested by a generalized estimating equation (p=0·03 and p<0·01, respectively); this was not observed for patients with SARS-CoV-2 and no secondary infection (p=0·26). Greater HLD-DR expression was associated with decreased odds of hospital mortality (odds ratio 0.99, 95% confidence interval 0.99-0.99, p=0·049); there were no significant associations between hospital mortality and other day 1 indicators of immune suppression, as listed in **Supplemental Table 3**.

### 3.3 MDSC Trajectories

**Figure 4:**
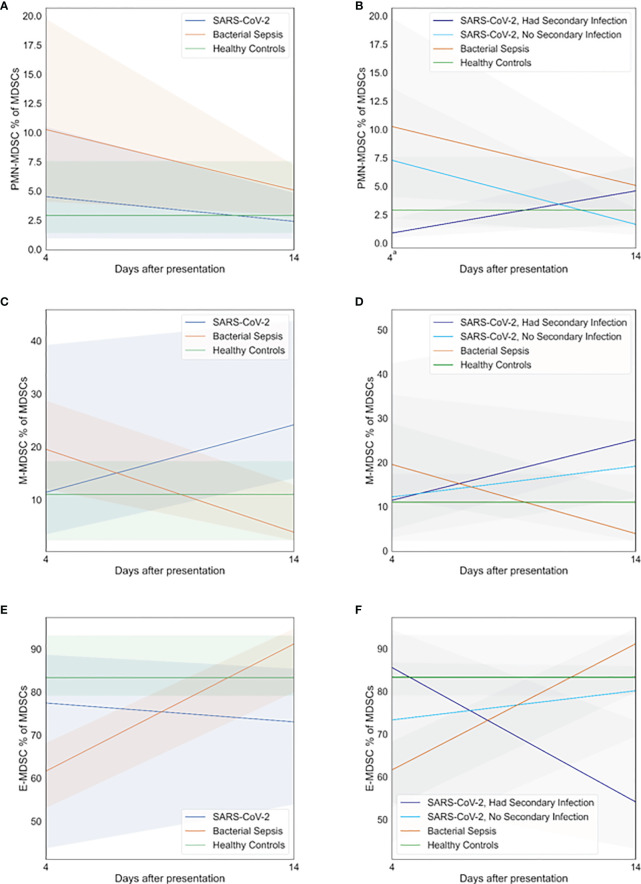
Myeloid-derived suppressor cell (MDSC) profiles. Patients with SARS-CoV-2 and secondary bacterial infection had myeloid-derived suppressor cell profiles similar to those of healthy controls. Subgroup analyses demonstrated that patients with SARS-CoV-2 who developed secondary bacterial infection had lower proportions of granulocytic (PMN)-MDCSs on day 4 compared with bacterial sepsis (p=0.05). All other proportions of PMN-MDSCs, monocytic (M)-MDSCs, and early progenitor (E)-MDSCs were similar among all groups at all time points. **(A)** PMN-MDSC on days 4 and 14, primary analysis. **(B)** PMN-MDSC on days 4 and 14, subgroup analysis. **(C)** M-MDSC on days 4 and 14, primary analysis. **(D)** M-MDSC on days 4 and 14, subgroup analysis. **(E)** E-MDSC on days 4 and 14, primary analysis. **(F)** E-MDSC on days 4 and 14, subgroup analysis. At each time point, all available values (as listed in **Supplemental Table 2**) were included in all statistical tests for 30 SARS-CoV-2 patients, 10 bacterial sepsis patients, and 10 healthy control patients. Groups were compared by generalized estimating equations with p-values corrected for multiple comparisons by the Benjamini-Hochberg procedure. a p≤0.05 for SARS-CoV-2 with secondary infection vs. Bacterial Sepsis.

The expansion of immunosuppressive MDSC populations in both SARS-CoV-2 and bacterial sepsis patients has been reported (15, 16), although they have only occasionally been compared directly over time. We examined MDSC expansion on days 4 and 14 after sepsis adjudication, as illustrated in **Figure 4**. Patients with SARS-CoV-2 and secondary bacterial infection had lower proportions of PMN-MDCSs on day 4 compared with bacterial sepsis (**Figure 4A**, p=0·05). All other proportions of PMN-, M-, and E-MDSCs were similar among all groups at all time points. In the SARS-CoV-2 patients who developed secondary bacterial infections, M-MDSCs decreased (**Figure 4D**) while E-MDSCs increased (**Figure 4F**) significantly over time, as tested by generalized estimating equations (p<0·01 and p=0·02, respectively). Although SARS-CoV-2 patients without secondary bacterial infection had no significant changes in MDSCs over time, among SARS-CoV-2 patients who developed a secondary bacterial infection, PMN-MDCSs increased (**Figure 4B**) while E-MDSCs decreased (**Figure 4F**) significantly over time (p=0·02 and p=0·04, respectively). There were no significant associations between hospital mortality and day 4 MDSCs, as listed in **Supplemental Table 3**.

## 4 Discussion

### 4.1 Summary of Major Findings

In this comparison of time-dependent immune dyscrasia in SARS-CoV-2 and bacterial sepsis, we observed that bacterial sepsis patients had early, severe inflammation, profound, comprehensive immune suppression, and life-threatening organ dysfunction that resolved over time while SARS-CoV-2 patients had less early inflammation and organ dysfunction but had persistent inflammation and immunosuppression and worse clinical outcomes. These inflammatory and immunologic phenomena were apparent in phenotypic blood leukocyte changes, plasma cytokine concentrations, and functional measures of innate and adaptive immunity.

### 4.2 Interpretation of Major Findings

There are several possible explanations for these phenomena, one being the underlying differences in host protective immune responses to viral and bacterial infections. Unlike bacteria, viral infections are recognized primarily by intracellular pattern recognition receptors, including TLR3, 7 and 9. Viral RNAs are additionally recognized by the intracellular RIG-1 and the cGAS/STING signaling system (17). These pathways lead ultimately to NF-kappa-B signaling, but signal predominantly through type I and type III interferons (18). Additionally, the initial target for SARS-CoV-2 infection is alveolar epithelial cells. Progressive disease in critical SARS-CoV-2 infections appears to require the interaction of infected epithelial cells with infiltrating immune cells, including macrophages and plasmacytoid dendritic cells. The local production of both cell-recruiting chemokines, such as CCL2, CCL3, CCL20, CXCL1, CXCL3, CXCL10, IL8, and the inflammatory cytokines, IL-1-beta and TNF-alpha further amplifies inflammation (19). These inflammatory mediators can subsequently damage the epithelial–endothelial barrier through macrophage infiltration, leading to progressive disease and host dissemination. Furthermore, these cell-to-cell interactions can persist for hours to days after the infection, resulting often in clinical disease progression despite declining viral titers (20, 21).

It also remains plausible that a major contributor to observed differences in SARS-CoV-2 and bacterial sepsis immune responses is more simple: bacterial sepsis patients received treatment with curative intent (i.e., early antibiotic therapy and source control of infection when possible) while SARS-CoV-2 patients received supportive care alone. Profound and comprehensive immunosuppression in both groups was evident by suppressed HLA-DR expression, reduced *ex vivo* IFN-gamma production, increased sPD-L1 concentrations, and expanded numbers of MDSCs. These findings suggest an increased vulnerability to secondary infections and the inability to resolve the initial septic insult without exogenous, curative treatment, which was available for bacterial sepsis but not for SARS-CoV-2 (22, 23).

### 4.3 Context From Prior Work

Most previous work has compared inflammation and immunosuppression between SARS-CoV-2 and bacterial sepsis at isolated, early time-points. An earlier study by our group (1) showed that patients with SARS-CoV-2 had a persistently suppressed functional immune response to *ex vivo* stimulation (ELISpot) that persisted for at least two weeks, although a direct comparison of the time dependent changes was not conducted with bacterial sepsis. In a comparison of 46 critically ill patients with bacterial sepsis and 21 critically ill patients with SARS-CoV-2 in China by Ren et al. (24), the bacterial sepsis cohort had higher initial SOFA and APACHE II scores, similar to our results. Unlike our results, mortality was significantly higher after bacterial sepsis (35% vs. 5%). Immune profiling was limited to absolute counts of cytotoxic T cells, helper T cells, and total T cell counts, each lower in the SARS-CoV-2 cohort; inflammation was approximated by C-reactive protein levels, which were higher in the SARS-CoV-2 cohort, which may account for differences in survival after bacterial sepsis. Additionally, initial bacterial sepsis illness severity was greater in the Ren (24) study, evident by higher APACHE II scores (17·0 vs. 12·5), and their clinical practices in managing bacterial sepsis were not described and may be different than ours. In a retrospective study by Dong et al. (25) of 64 bacterial sepsis patients and 43 patients with SARS-CoV-2 complicated by the acute respiratory distress syndrome, the number of cytotoxic T cells, helper T cells, and total T cells were similar between groups; early inflammatory cytokine levels were higher in the bacterial sepsis group, similar to our results. Immune function was assayed by enumerating PMA/ionomycin-stimulated IFN-γ positive T and NK cells, which were similar between groups. Clinical outcomes were not presented. Similarly, Monneret and colleagues (16) examined MDSC expansion in both SARS-CoV-2 and bacterial sepsis, and observed expansion of predominantly PMN-MDSCs beginning 3-5 days after sepsis. They classified whole blood MDSCs based on LOX-1 expression, identifying PMN-MDSCs as being the LOX-1^+^ subset of CD45^dim^, side scatter^high^ leukocytes. In contrast, we used the more traditional gating strategy HLA-DR^-/dim^, CD11b^+^, CD33^+^ and CD66b^+^ expression from the PBMC fraction (26), capturing the low-density fraction of PMN-MDSCs, and then confirming that more than 80% of these cells were LOX-1^+^. In our study, both SARS-CoV-2 and bacterial sepsis were associated with increased expansion of both M- and PMN-MSDCs and reduced number of E-MDSCs.

Importantly, *in vivo* evidence of increased inflammation, documented by elevated cytokine concentrations, does not necessarily imply an increased inflammatory capability. Although Karki et al. (27) found that TNF-alpha and IFN-gamma concentrations were elevated in serious COVID19 infections and *ex vivo* were associated with increased programmed death of inflammatory cells and mortality, the authors did not look at host capability to produce cytokines. Over three decades ago, Munoz and Cavaillon demonstrated that blood monocytes from septic patients when stimulated *ex vivo* with endotoxin produced less TNF, IL-1 and IL-6 than monocytes from healthy controls, and non-survivors produced even less (28). The findings reported here are not inconsistent with either Karki or Munoz and demonstrate that in the presence of elevated proinflammatory cytokines, the capacity of cells to produce these inflammatory mediators in response to *ex vivo* stimulation is suppressed, consistent with simultaneous immune suppression.

In the most robust prior comparison of immune function in SARS-CoV-2 versus bacterial sepsis, Reyes et al. (29) used eight publicly available, transcriptomic datasets representing 1,013 patients with SARS-CoV-2 or bacterial sepsis and obtained additional plasma samples from four SARS-CoV-2 and four bacterial sepsis patients; scRNA-seq data was available for all subjects apart from one study by Sweeney et al. (30) containing bulk transcriptomics from whole blood of 861 bacterial sepsis patients. In a series of elegant experiments evaluating CD14^+^ monocyte expansion, gene expression associations with sepsis severity, myelopoiesis induction in healthy monocytes by plasma from bacterial sepsis or COVID-19 patients, and the role of IL-6 and IL-10 in enhancing CD14^+^ monocyte expansion, the authors demonstrate that CD14^+^ monocytes were profoundly immunosuppressive and induced by inflammatory cytokines.

One of the major controversies in sepsis immunotherapy is whether immunosuppression is adaptive or maladaptive during severe inflammation, and therefore, how targeted immunomodulators should augment or attenuate inflammatory and immunosuppressive pathways (31–34). Our results suggest that these questions cannot be answered fully without deep understanding of changes in inflammatory and immunosuppressive pathways over time. In addition, the mere presence of lymphocytes and lymphocyte progenitors, as determined by cellular surface markers, cannot elucidate the pathophysiology of sepsis; functional assays, such as ELISpot, offer greater insight regarding immune cell function (14, 35). Therefore, we suggest that functional assays are essential components of sepsis immunotherapy trials. In addition, based on evidence that there are distinct sepsis phenotypes with unique responses to both standard clinical management strategies and immune modulators, we suggest that sepsis trials should target specific endotypes and subendotypes (8). Finally, previous work suggests that the primary pathophysiology of COVID-19 may be profound immunosuppression, rather than an early, inflammatory cytokine storm. Our present and prior work argues against an early cytokine storm in SARS-CoV-2 (2), but rather a late cytokine response associated with progressive disease (1). Evidence from a human, observational study casts doubt on the utility of anti-TNF-alpha therapy for SARS-CoV-2 infection (36). Yet, emerging evidence suggests potential utility for IL-1 and IL-6 inhibitors (37, 38). Collectively, it appears that both immunosuppression and persistent inflammation contribute to the pathophysiology of SARS-CoV-2 infection, and may represent therapeutic targets.

### 4.4 Limitations

This study was limited by its single-institution design, limiting generalizability to other practice settings, and by its small sample sizes, which precluded analysis of sepsis endotypes and sub-endotypes. In addition, immune profiling is presented alongside clinical outcomes to demonstrate associations but not causality. SARS-CoV-2-related coagulopathy and lung-protective strategies may have substantial effects on clinical outcomes, as do the timing and efficacy of appropriate antibiotic therapy and source control for bacterial sepsis; none of these variables are presented herein (3, 39–42). Future research should include these variables in a larger, multi-center effort that maintains the potential benefits of dynamic assessments of immune cell function in relation to clinical outcomes.

### 4.5 Conclusions

Bacterial sepsis patients had early, severe inflammation, profound immune suppression, and life-threatening organ dysfunction that resolved over time; SARS-CoV-2 patients had less severe early inflammation and organ dysfunction but had persistent inflammation and immunosuppression and suffered worse clinical outcomes, especially when SARS-CoV-2 infection was followed by secondary bacterial infection. These observations must be interpreted in the context that the host responses to viral and bacterial sepsis differ substantially, and that bacterial sepsis patients received treatment with curative intent while SARS-CoV-2 patients received supportive care alone. Profound and comprehensive immunosuppression in both groups was evident by suppressed HLA-DR expression, reduced IFN-gamma production, and increased sPD-L1 concentrations, suggesting vulnerability to secondary infections and the inability to resolve the initial septic insult without exogenous, curative treatment.

## Data Availability Statement

All de-identified data used in the analyses presented herein and an accompanying data dictionary will be available upon publication *via* the University of Florida Clinical and Translational Science Institute Biorepository with a signed data access agreement.

## Ethics Statement

The studies involving human participants were reviewed and approved by University of Florida Institutional Review Board. The patients/participants provided their written informed consent to participate in this study.

## Author Contributions

SB and LLM conceived the study, oversaw the data analysis and edited the manuscript. TJL wrote the manuscript, finalized the figures and tables, and takes full responsibility for the integrity of the data analysis. LLM has also verified the underlying data. LZ and RB provided statistical analysis and study design. RU, MD and PS conducted and oversaw the analyses. TS, MM, KR, RH and OL analyzed the data and edited the manuscript.

## Funding

Research reported in this publication was supported by the University of Florida Clinical and Translational Science Institute, which is supported in part by the NIH National Center for Advancing Translational Sciences under award number UL1 TR001427. T.J.L. was supported by the National Institute of General Medical Sciences (NIGMS) of the National Institutes of Health under Award Number K23 GM140268. Infrastructure support was also provided by grants RM1 GM139690-01 and R01 GM132364-01 from NIGMS. Funding sources had no role in study design, in the collection, analysis, and interpretation of data, in the writing of the report, or in the decision to submit the paper for publication.

## Author Disclaimer

The content is solely the responsibility of the authors and does not necessarily represent the official views of the National Institutes of Health.

## Conflict of Interest

The authors declare that the research was conducted in the absence of any commercial or financial relationships that could be construed as a potential conflict of interest.

## Publisher’s Note

All claims expressed in this article are solely those of the authors and do not necessarily represent those of their affiliated organizations, or those of the publisher, the editors and the reviewers. Any product that may be evaluated in this article, or claim that may be made by its manufacturer, is not guaranteed or endorsed by the publisher.
