# Supplementary material for: Overlapping but Disparate Inflammatory and Immunosuppressive Responses to SARS-CoV-2 and Bacterial Sepsis: An Immunological Time Course Analysis
Source: Front Immunol. 2021 Dec 9;12:792448. doi: 10.3389/fimmu.2021.792448 (PMC8696010; doi:10.3389/fimmu.2021.792448)
Supplement: Supplementary file 1 [file DataSheet_1.docx]

Supplementary Material

**Table of Contents**

**Supplemental Table 1.** STROBE checklist for cross-sectional studies..…………...………Page 2

**Supplemental Table 2.** Summary of missing data………………………....……….………Page 3

**Supplemental Table 3.** Univariate associations between hospital mortality and early indicators of inflammation, immune suppression, and myeloid derived suppressor cell (MDSC) populations……………………………………….………………………....……….………Page 6

**Supplemental Figure 1.** Gating strategy for myeloid-derived suppressor cells…………….Page 7

**Supplemental Figure 2.** Illustrative examples of enzyme-linked immunospot assays….….Page 8

**Supplemental Figure 3.** Additional inflammatory cytokine time series analyses…………..Page 9

**Supplemental Table 1.** STROBE checklist for cross-sectional studies.

|  | Item No | Recommendation | Page No |
| --- | --- | --- | --- |
| **Title and abstract** | 1 | (*a*) Indicate the study’s design with a commonly used term in the title or the abstract | 1 |
|  |  | (*b*) Provide in the abstract an informative and balanced summary of what was done and what was found | 1 |
| Introduction | | | |
| Background/rationale | 2 | Explain the scientific background and rationale for the investigation being reported | 2 |
| Objectives | 3 | State specific objectives, including any prespecified hypotheses | 2 |
| Methods | | | |
| Study design | 4 | Present key elements of study design early in the paper | 2-4 |
| Setting | 5 | Describe the setting, locations, and relevant dates, including periods of recruitment, exposure, follow-up, and data collection | 2-4 |
| Participants | 6 | (*a*) Give the eligibility criteria, and the sources and methods of selection of participants | 2,3 |
| Variables | 7 | Clearly define all outcomes, exposures, predictors, potential confounders, and effect modifiers. Give diagnostic criteria, if applicable | 2-4 |
| Data sources/ measurement | 8 | For each variable of interest, give sources of data and details of methods of assessment (measurement). Describe comparability of assessment methods if there is more than one group | 2-4 |
| Bias | 9 | Describe any efforts to address potential sources of bias | 3, 4 |
| Study size | 10 | Explain how the study size was arrived at | 4 |
| Quantitative variables | 11 | Explain how quantitative variables were handled in the analyses. If applicable, describe which groupings were chosen and why | 4 |
| Statistical methods | 12 | (*a*) Describe all statistical methods, including those used to control for confounding | 4 |
|  |  | (*b*) Describe any methods used to examine subgroups and interactions | 4 |
|  |  | (*c*) Explain how missing data were addressed | 4 |
|  |  | (*d*) If applicable, describe analytical methods taking account of sampling strategy | Not applicable |
|  |  | (*e*) Describe any sensitivity analyses | 4 |
| Results | | | |
| Participants | 13 | (a) Report numbers of individuals at each stage of study—eg numbers potentially eligible, examined for eligibility, confirmed eligible, included in the study, completing follow-up, and analysed | 4 |
|  |  | (b) Give reasons for non-participation at each stage | 4 |
|  |  | (c) Consider use of a flow diagram | Considered |
| Descriptive data | 14 | (a) Give characteristics of study participants (eg demographic, clinical, social) and information on exposures and potential confounders | 4, 5, Table 1 |
|  |  | (b) Indicate number of participants with missing data for each variable of interest | Supplement |
| Outcome data | 15 | Report numbers of outcome events or summary measures | 4-6, Table 2 |
| Main results | 16 | (*a*) Give unadjusted estimates and, if applicable, confounder-adjusted estimates and their precision (eg, 95% confidence interval). Make clear which confounders were adjusted for and why they were included | 4-6, Table 2 |
|  |  | (*b*) Report category boundaries when continuous variables were categorized | Not applicable |
|  |  | (*c*) If relevant, consider translating estimates of relative risk into absolute risk for a meaningful time period | Not applicable |
| Other analyses | 17 | Report other analyses done—eg analyses of subgroups and interactions, and sensitivity analyses | 5, 6, Figures 2 and 4 |
| Discussion | | | |
| Key results | 18 | Summarise key results with reference to study objectives | 6 |
| Limitations | 19 | Discuss limitations of the study, taking into account sources of potential bias or imprecision. Discuss both direction and magnitude of any potential bias | 8 |
| Interpretation | 20 | Give a cautious overall interpretation of results considering objectives, limitations, multiplicity of analyses, results from similar studies, and other relevant evidence | 6-8 |
| Generalisability | 21 | Discuss the generalisability (external validity) of the study results | 8 |
| Other information | | | |
| Funding | 22 | Give the source of funding and the role of the funders for the present study and, if applicable, for the original study on which the present article is based | 8 |

**Supplemental Table 2.** Summary of data missing at random or censored over time relative to the total number of subjects enrolled.

| Variable | Percent missing |
| --- | --- |
| Age | 0.0 |
| Female | 0.0 |
| Race |  |
| African American | 0.0 |
| American Indian | 0.0 |
| Asian | 0.0 |
| Pacific Islander | 0.0 |
| White | 0.0 |
| Other or Unknown | 0.0 |
| Body mass index | 0.0 |
| Charlson comorbidity index | 0.0 |
| Comorbidities |  |
| Acute renal failure | 0.0 |
| Chronic obstructive pulmonary disease | 0.0 |
| Congestive heart failure | 0.0 |
| Current smoker | 0.0 |
| Diabetes | 0.0 |
| Oral hypoglycemic prescription | 0.0 |
| Insulin prescription | 0.0 |
| Dialysis dependent | 0.0 |
| Disseminated cancer | 0.0 |
| Hypertension | 0.0 |
| Myocardial infarction | 0.0 |
| Peripheral vascular disease | 0.0 |
| Steroid use | 0.0 |
| >10% weight loss in prior 6 months | 0.0 |
| Illness severity |  |
| APACHE II score | 0.0 |
| MEWS score | 0.0 |
| Received vasopressors | 0.0 |
| Received mechanical ventilation | 0.0 |
| Secondary infection | 0.0 |
| Pulmonary infection | 0.0 |
| Bloodstream infection | 0.0 |
| Skin or soft tissue infection | 0.0 |
| Non-infectious complication | 0.0 |
| Days between onset and secondary infection | 0.0 |
| ICU length of stay | 0.0 |
| ICU length of stay ≥14 days | 0.0 |
| ICU-free days | 0.0 |
| Ventilator days | 0.0 |
| Hospital length of stay | 0.0 |
| Discharge disposition |  |
| Hospital mortality | 0.0 |
| Home | 0.0 |
| Hospice | 0.0 |
| Inpatient rehabilitation | 0.0 |
| Left against medical advice | 0.0 |
| Long term acute care | 0.0 |
| Skilled nursing facility | 0.0 |
| Poor discharge disposition | 0.0 |
| Chronic critical illness | 0.0 |
| Adverse clinical outcome | 0.0 |
| Discharge to 6-month follow-up |  |
| Readmission | 15.0 |
| Infection without readmission | 15.0 |
| Death | 15.0 |
| 6-12-month follow-up |  |
| Readmission | 62.5 |
| Infection without readmission | 62.5 |
| Death | 62.5 |
| Day 1 analytes |  |
| IL-6 | 0.0 |
| TNF-alpha | 0.0 |
| IL-8/CXCL-8 | 0.0 |
| IL-1 beta/IL-1F2 | 0.0 |
| HLA-ABC | 27.5 |
| PD-L1 1/B7-H1 | 0.0 |
| IFN-gamma spot count, with IL-7 stimulation | 5.0 |
| IFN-gamma spot size, with IL-7 stimulation | 5.0 |
| IFN-gamma expression, with IL-7 stimulation | 5.0 |
| IFN-gamma spot count, without IL-7 stimulation | 5.0 |
| IFN-gamma spot size, without IL-7 stimulation | 5.0 |
| IFN-gamma expression, without IL-7 stimulation | 5.0 |
| TNF-alpha spot count, with IL-7 stimulation | 7.5 |
| TNF-alpha spot size, with IL-7 stimulation | 7.5 |
| TNF-alpha expression, with IL-7 stimulation | 7.5 |
| TNF-alpha spot count, without IL-7 stimulation | 7.5 |
| TNF-alpha spot size, without IL-7 stimulation | 7.5 |
| TNF-alpha expression, without IL-7 stimulation | 7.5 |
| Day 4 analytes |  |
| IL-6 | 12.5 |
| TNF-alpha | 12.5 |
| IL-8/CXCL-8 | 12.5 |
| IL-1 beta/IL-1F2 | 12.5 |
| HLA-ABC | 20.0 |
| PD-L1 1/B7-H1 | 12.5 |
| IFN-gamma spot count, with IL-7 stimulation | 22.5 |
| IFN-gamma spot size, with IL-7 stimulation | 22.5 |
| IFN-gamma expression, with IL-7 stimulation | 22.5 |
| IFN-gamma spot count, without IL-7 stimulation | 22.5 |
| IFN-gamma spot size, without IL-7 stimulation | 22.5 |
| IFN-gamma expression, without IL-7 stimulation | 22.5 |
| TNF-alpha spot count, with IL-7 stimulation | 25.0 |
| TNF-alpha spot size, with IL-7 stimulation | 25.0 |
| TNF-alpha expression, with IL-7 stimulation | 25.0 |
| TNF-alpha spot count, without IL-7 stimulation | 25.0 |
| TNF-alpha spot size, without IL-7 stimulation | 25.0 |
| TNF-alpha expression, without IL-7 stimulation | 25.0 |
| PMN-MDSCs | 12.5 |
| M-MDSCs | 12.5 |
| E-MDSCs | 12.5 |
| Day 7 analytes |  |
| IL-6 | 40.0 |
| TNF-alpha | 40.0 |
| IL-8/CXCL-8 | 40.0 |
| IL-1 beta/IL-1F2 | 40.0 |
| HLA-ABC | 52.5 |
| PD-L1 1/B7-H1 | 40.0 |
| IFN-gamma spot count, with IL-7 stimulation | 42.5 |
| IFN-gamma spot size, with IL-7 stimulation | 42.5 |
| IFN-gamma expression, with IL-7 stimulation | 42.5 |
| IFN-gamma spot count, without IL-7 stimulation | 42.5 |
| IFN-gamma spot size, without IL-7 stimulation | 42.5 |
| IFN-gamma expression, without IL-7 stimulation | 42.5 |
| TNF-alpha spot count, with IL-7 stimulation | 40.0 |
| TNF-alpha spot size, with IL-7 stimulation | 40.0 |
| TNF-alpha expression, with IL-7 stimulation | 40.0 |
| TNF-alpha spot count, without IL-7 stimulation | 40.0 |
| TNF-alpha spot size, without IL-7 stimulation | 40.0 |
| TNF-alpha expression, without IL-7 stimulation | 40.0 |
| Day 14 analytes |  |
| IL-6 | 65.0 |
| TNF-alpha | 65.0 |
| IL-8/CXCL-8 | 65.0 |
| IL-1 beta/IL-1F2 | 65.0 |
| PD-L1 1/B7-H1 | 65.0 |
| PMN-MDSCs | 70.0 |
| M-MDSCs | 70.0 |
| E-MDSCs | 70.0 |
| Day 21 analytes |  |
| IL-6 | 87.5 |
| TNF-alpha | 87.5 |
| IL-8/CXCL-8 | 87.5 |
| IL-1 beta/IL-1F2 | 87.5 |
| PD-L1 1/B7-H1 | 87.5 |

**Supplemental Table 3.** Univariate associations between hospital mortality and early indicators of inflammation, immune suppression, and myeloid derived suppressor cell (MDSC) populations among critically ill subjects with bacterial sepsis or SARS-Cov-2 infection.

| Analytes | Odds Ratio | P-value |
| --- | --- | --- |
| Day 1 IL-1 beta | 0.88 (0.41 - 1.08) | 0.647 |
| Day 1 IL-6 | 1.00 (0.98 - 1.00) | 0.569 |
| Day 1 IL-8 | 1.00 (0.97 - 1.02) | 0.945 |
| Day 1 IL-10 | 0.98 (0.87 - 1.05) | 0.619 |
| Day 1 TNF-alpha | 1.02 (0.88 - 1.13) | 0.710 |
| Day 1 G-CSF | 1.00 (0.97 - 1.00) | 0.735 |
| Day 1 MCP-1/CCL2 | 1.00 (1.00-1.00) | 0.529 |
| Day 1 HLA-DR expression | 0.99 (0.99-0.99) | **0.049** |
| Day 1 sPD-L1 | 1.01 (1.00 - 1.02) | 0.076 |
| Day 1 TNF-alpha LPS spot count | 1.00 (1.00 - 1.01) | 0.175 |
| Day 1 TNF-alpha LPS + IL-7 spot count | 1.01 (1.00 - 1.01) | 0.070 |
| Day 1 TNF-alpha LPS spot size | 1.00 (0.82 - 1.18) | 0.991 |
| Day 1 TNF-alpha LPS + IL-7 spot size | 1.01 (0.87 - 1.16) | 0.869 |
| Day 1 TNF-alpha LPS expression | 1.00 (1.00 - 1.00) | 0.174 |
| Day 1 TNF-alpha LPS + IL-7 expression | 1.00 (1.00 - 1.00) | 0.109 |
| Day 1 IFN-gamma CD3/28 spot count | 1.00 (1.00 - 1.01) | 0.427 |
| Day 1 IFN-gamma CD3/28 + IL-7 spot count | 1.00 (1.00 - 1.01) | 0.396 |
| Day 1 IFN-gamma CD3/28 spot size | 1.05 (0.73 - 1.52) | 0.786 |
| Day 1 IFN-gamma CD3/28 + IL-7 spot size | 1.02 (0.76 - 1.33) | 0.908 |
| Day 1 IFN-gamma CD3/28 expression | 1.00 (1.00 - 1.00) | 0.527 |
| Day 1 IFN-gamma CD3/28 + IL-7 expression | 1.00 (1.00 - 1.00) | 0.464 |
| Day 4 M-MDSC % of MDSCs | 1.02 (0.98 - 1.06) | 0.239 |
| Day 4 PMN-MDSC % of MDSCs | 0.92 (0.72 - 1.02) | 0.294 |
| Day 4 E-MDSC % of MDSCs | 0.99 (0.95 - 1.03) | 0.557 |

IL: interleukin, TNF: tumor necrosis factor, G-CSF: granulocyte-colony stimulating factor, MCP: monocyte chemoattractant protein, CCL: chemokine ligand, HLA: human leukocyte antigen, PD-L: programmed death-ligand, LPS: lipopolysaccharide, M-MDSCs: monocytic MDSCs, PMN-MDSCs: granulocytic MDSCs, E-MDSCs: erythroid MDSCs. P-values were not corrected for multiple comparisons.


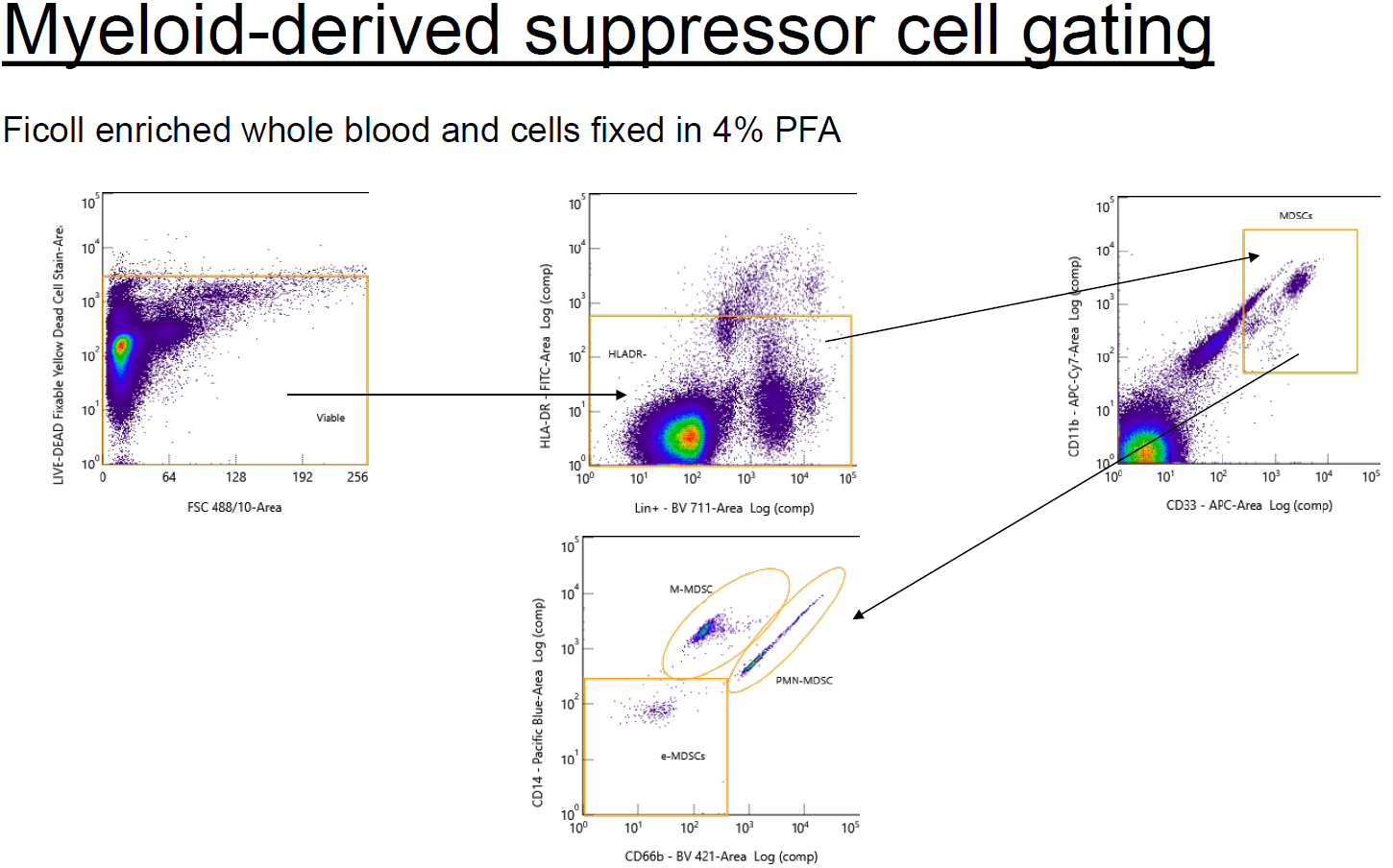


**Supplemental Figure 1.** Gating strategy for myeloid-derived suppressor cells with Ficoll enriched whole blood and cells fixed in 4% paraformaldehyde solution.

**
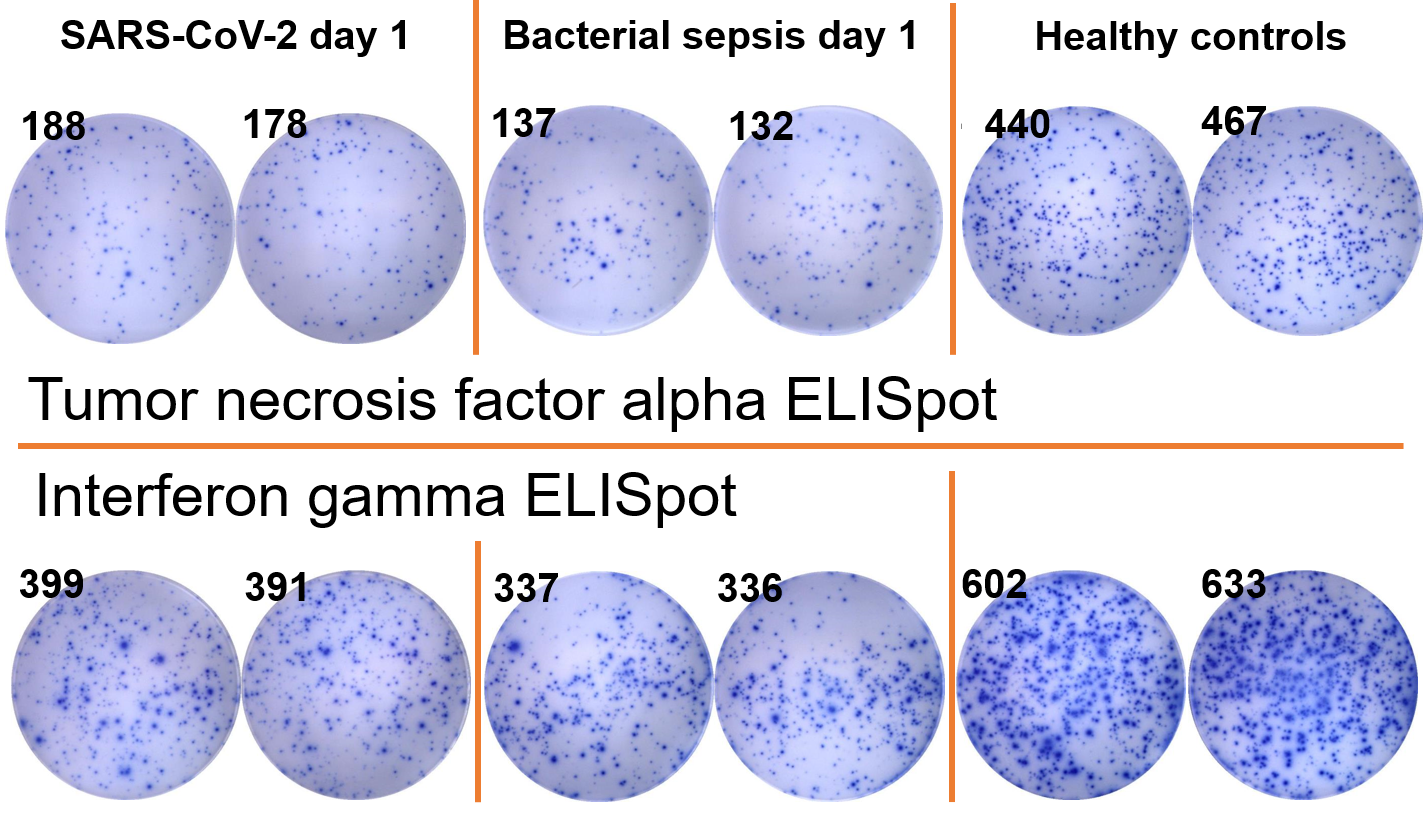
**

**Supplemental Figure 2.** Illustrative examples of enzyme-linked immunospot assays (ELISpot). Numbers appearing on the top left of each well represent spot counts.


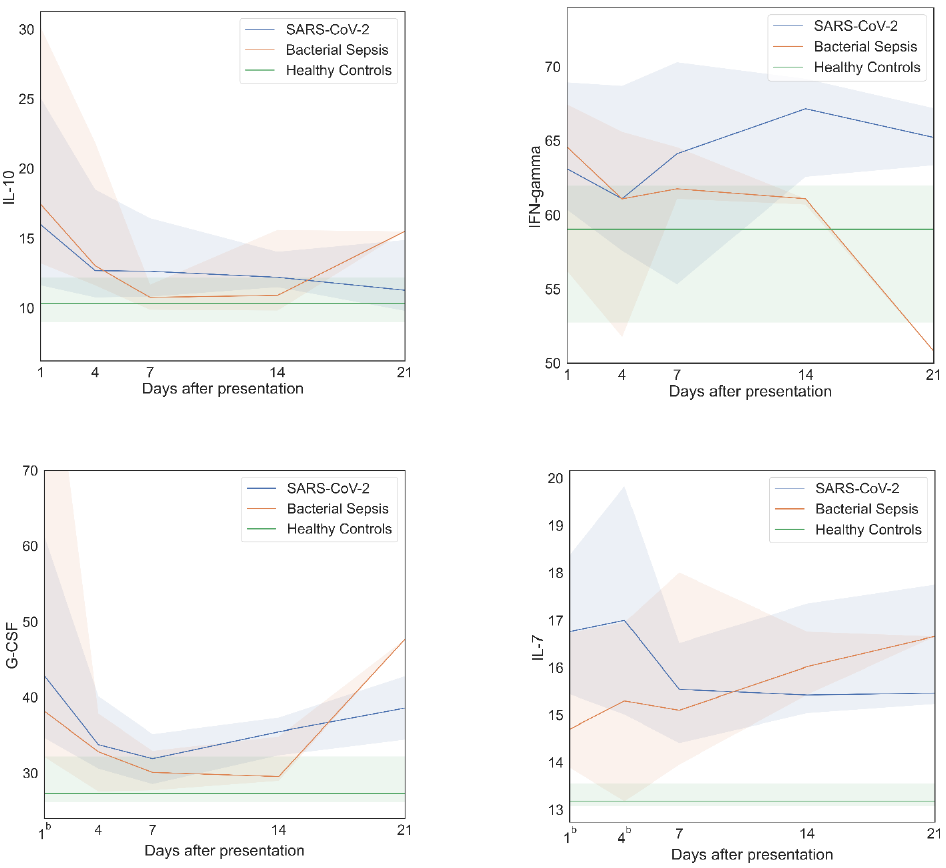


**Supplemental Figure 3.** Additional inflammatory cytokine time series analyses. After bacterial sepsis, inflammatory cytokines peaked on sampling day one and then declined; after SARS-CoV-2 infection, inflammatory cytokines were persistently elevated or increased over time. Data are presented as median values (colored lines) and interquartile ranges (shaded regions). Groups were compared by generalized estimating equations with p-values corrected for multiple comparisons by the Benjamini-Hochberg procedure. Superscript letters indicate time points at which group comparisons had p≤0.05. At each time point, all available values (as listed in **Supplemental Table 2**) were included in all statistical tests for 30 SARS-CoV-2 patients, 10 bacterial sepsis patients, and 10 healthy control patients. ^a^SARS-CoV-2 vs. Bacterial Sepsis; ^b^SARS-CoV-2 vs. Healthy Controls; ^c^Bacterial Sepsis vs. Healthy Controls. IL: interleukin, IFN: interferon, G-CSF: granulocyte colony-stimulating factor.
